# Impact of vacuum ultraviolet (VUV) photolysis on ethylene degradation kinetics and removal in mixed-fruit storage, and direct exposure to ‘Fuji’ apples during storage

**DOI:** 10.1007/s13197-023-05775-3

**Published:** 2023-06-07

**Authors:** Bongolwethu P. Mabusela, Zinash A. Belay, Buntu Godongwana, Oluwafemi James Caleb

**Affiliations:** 1 Agri-Food Systems and Omics Laboratory, Post-Harvest and Agro-Processing Technologies (PHATs), Agricultural Research Council (ARC) Infruitec-Nietvoorbij, Stellenbosch, 7599 South Africa; 2 Department of Chemical Engineering, Cape Peninsula University of Technology, P.O Box 1906, Bellville, 7535 South Africa; 3 Department of Horticultural Science, Faculty of AgriSciences, Stellenbosch University, Private Bag X1, Matieland, 7602 South Africa; 4 Africa Institute for Postharvest Technology, Faculty of AgriSciences, Stellenbosch University, Private Bag X1, Matieland, 7602 South Africa

**Keywords:** Fresh fruit, Respiration rate, Postharvest value chains, Shelf-life extension

## Abstract

Accumulated ethylene in fruit storage/transportation causes rapid senescence resulting in reduced shelf-life and postharvest losses. The aim of this study was to investigate the application of vacuum ultraviolet (VUV) photolysis modular reactor for fruit storage. The first experiment compared the effectiveness of VUV photolysis reactor with the standard fruit industry adsorbent (potassium permanganate, KMnO_4_) on the removal of ethylene from mixed-fruit loading of apples, banana, and pears stored at ambient temperature (16 °C) for 6 days. Second study evaluated the impact of direct VUV radiation on quality attributes of apples stored at 10 °C for 21 days. Results showed that ethylene produced in mixed-fruit loading storage significantly (*p* < 0.05) reduced by 86.9% in the storage chamber connected to VUV modular reactor compared to 25.4% for storage under potassium permanganate. Direct exposure of apples to VUV radiation successfully reduced both ethylene and respiration rate but damaged the skin of the apples. Hue angle and lightness (L*) for apples exposed to VUV radiation declined significantly (*p* < 0.05) from 60.7 ± 1.09 to 33.5 ± 9.51 and 58.1 ± 3.60 to 50.4 ± 1.13, respectively. This study showed the potential of VUV photolysis as an innovative technique for removing ethylene from storage facility.

## Introduction

The response of harvested fruit to endogenously produced and exogenously applied ethylene are numerous and varied (Palou et al. 2003). The impact of ethylene on fresh fruit could be considered beneficial or detrimental depending on the extent of exposure and the type of product. The presence of ethylene even at low concentrations (as low as 1 mg/kg) could induce precocious fruit ripening. Some of the detrimental impacts of ethylene include acceleration of physiological disorders, excessive softening, over-ripening and colour change leading to a reduction in postharvest life. Thus, the removal of ethylene from storage systems is of paramount importance in the horticultural industry. Improving conventional techniques and developing new techniques for the control of ethylene during the transport and storage period has proven to be challenging (Pathak et al. 2017a).

Conventional ethylene removal postharvest strategies include air ventilation, low-temperature storage and controlled atmosphere (CA) storage (Mabusela et al. 2021b). Other techniques include the use of different adsorber/absorbers, the use of potassium permanganate (KMnO_4_) (Aprilliani et al. 2018), ozone and catalytic oxidation (Smilanick 2003). The use of adsorbing/absorbing materials such as zeolites and activated carbon, and oxidizers such as KMnO_4_ are not suitable for long term storage and distant transportation because these materials saturate rapidly thereby necessitating frequent replenishment. Moreover, potassium permanganate oxidation results in toxic residues that require further disposal (Duque et al. 2021; Zhu et al. 2019). Catalytic oxidation, on the other hand, requires high temperatures rendering the technique energy-intensive (Keller et al. 2013). Meanwhile, some studies have shown the efficiency of photocatalytic oxidation (PCO) for ethylene oxidation to be dependent on carbon dioxide and water (Basso et al. 2018; Pathak et al. 2017b, 2019). While PCO has found great application in postharvest management, there are still inherent drawbacks that limit its commercialization such as the recombination of hole and electron which results in the reduction of oxidized species, and its declined removal efficiency at high relative humidity caused by the competing water and ethylene molecules for active sites (Pathak et al. 2017a).

Vacuum ultraviolet photolysis (VUV) is an emerging technique for ethylene postharvest management. Photolysis generally employs UV light sources, such as low-pressure and medium-pressure mercury lamps with approximately 85% output UV light at 254 nm and 15% output at 185 nm. The high energy photons generated at 185 nm are self-sufficient in decomposing oxygen and water molecules present in the air to produce highly reactive species such as atomic O_2_, hydroxyl radicals and ozone which are responsible for the oxidation of ethylene to carbon dioxide and water (Mabusela et al. 2021a). The application of VUV photolysis has been commonly used for the removal of organics in the aqueous phase and air pollutants (Huang et al. 2016a; Kang et al. 2018; Mahmoudkhani et al. 2016). This technique has demonstrated promising ethylene removal capabilities and was successful in prolonging the shelf-life of apples and kiwifruit (Pathak et al. 2017b).

However, the application of VUV photolysis for postharvest ethylene management is still very limited. The efficiency of this technique is dependent on the generated hydroxyl radicals that are responsible for the oxidation of ethylene to carbon dioxide and water (Mabusela et al. 2021a). Furthermore, the impact of the radicals and/or direct VUV radiation on the surface and/or quality of treated fruit is still not known. Thus, the objectives of this study are two-fold: (i) to investigate the ethylene degradation performance of a modular VUV photolysis reactor in comparison to the use of potassium permanganate under a mixed-fruit storage condition at 15 °C for 6 days; and (ii) to evaluate the effect of direct exposure to VUV radiation on the physiological parameters of apples under cool retail condition at 10 °C for 21 days.

## Materials and methods

### Plant material

All fruit samples used in this were obtained at commercial maturity from a fresh fruit retail market farm, Stellenbosch, South Africa. Fruit samples were transported under cool condition and in ventilated vehicle to the Agri-Food Systems and Omics Laboratory, Agricultural Research Council (ARC) Infruitec-Nietvoorbij, Stellenbosch, South Africa. On arrival, samples were sorted carefully to ensure uniformness and eliminate damaged or decayed fruit. Fruit surfaces were disinfected by dipping in NaOCl solution (≈ 200 mg/L) and stored at 5 °C before to the start of the full.

### VUV system setup

**Fig. 1:**
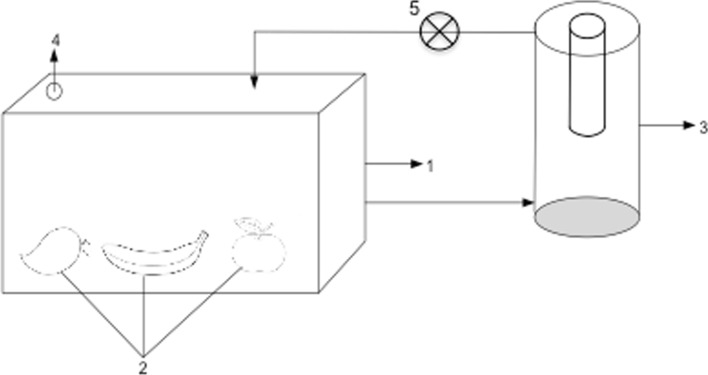
Schematic representation of the experimental setup used for the photolytic oxidation of ethylene emitted in the storage of mixed fruits. (1) Storage container; (2) fruits (banana, apples, and pears); (3) photolytic reactor (same as used in kinetic studies); (4) sample gas collection; and (5) circulating pump

The photolysis reactions were carried out using an in-house designed batch flow-type reactor system, similar to the one reported by Pathak et al. (2017b). The system consists of a cylindrical unit made of stainless steel (diameter = 12 cm, height 11 cm), and fitted with inlet and outlet ports at the top and bottom, respectively. The lid of the reactor was made of acrylic sheet and had an opening for the electric fittings of the VUV lamp. An ozone-producing VUV lamp with a power input of 3 W (Dinies, Villingendorf, Germany) was placed along the central axis of the reactor. The major emission of the irradiation by the lamp was at 254 nm, while minor emission (5—8%) was at 185 nm. A modular pump was used to circulate the air from the storage chamber and through the VUV photolysis reactor system as annotated in Fig. 1.

### Experiment I: ethylene degradation rate

Study was conducted to understand the impact of initial ethylene concentration on the efficiency of the reactor. Desired concentrations of ethylene were obtained by mixing ethylene from ethylene standard (100 mg/kg) with synthetic air. Initial ethylene concentrations investigated were 7, 55 and 67 mg/kg. Ethylene samples were taken at regular intervals from the reactor using an ICA 56-ethylene analyser (Fricaval 89 S.L, Valencia, Spain), which has an inlet and out-let pump that returned the gas sample to the reactor to ensure gas volume remained constant. All the experiments were carried out at atmospheric pressure and ambient room temperature. The photolytic efficiency was calculated using the following equation:

1 $$Ethylene removal \left( \% \right) = \frac{{C_{0} - C_{t} }}{{C_{0} }} \times 100$$

where C_0_ is the initial ethylene concentration (mg/kg), and C_t_ is the real-time concentration of ethylene during the photooxidation.

To determine the reaction kinetics of the photolysis reaction, a first-order kinetic model (Eq. 2) was assumed (Mortazavian et al. 2019):

2 $$ln\left( C \right) = ln\left( {C_{0} } \right) - kt$$

where, *t* is the reaction time (min), *C*_*0*_ and *C* (mg/kg) are the initial ethylene concentration and ethylene concentration at time t, respectively, and *k* is the first-order reaction rate constant (min^−1^).

### Degradation of ethylene during mixed-fruit storage

To evaluate the photolytic degradation of ethylene produced by mixed-fruits, three types of fruit apples, bananas and pears were selected for this study. The mixed-fruit were separated into three groups and placed in a 13 L storage chamber. The first group consisted of mixed-fruit stored without any ethylene removal strategy, which served as a control. The second group consisted of mixed-fruit stored with 10% KMnO_4_ (kg/kg of total fruit) deposited on a petri dish hanging in the center of the container to represent the industry practice. The third group consisted of the mixed-fruits storage chamber connected to the VUV photolysis reactor for continuous removal of ethylene (treatment) as shown in Fig. 1. This set-up was replicated in triplicate. The experiments were conducted at ambient winter room temperature averaging at 15 °C for 6 days. The ethylene concentration in the gas phase inside the chambers was monitored daily using ICA 56-ethylene analyser (Fricaval 89 S.L, Valencia, Spain).

### Experiment II: direct VUV treatment and storage conditions

Storage investigation of ‘Fuji’ apples was conducted using a 30 L plastic chamber fully covered in aluminum foil to prevent light from reacting with the plastic and fitted with three ozone-producing VUV lamps (Dinies, Villingendorf, Germany), with a power input of 3 W each. A total of 24 ‘Fuji’ apples were divided into two treatment groups of 12 samples. The first treatment group consisted of apples that were exposed directly to VUV light (approximately 15 cm away from the fruit), to understand the impact of direct exposure (mimicking retail markets, where apples a placed directly under UV light); while the second treatment did not have any VUV light (control). The storage chambers were not hermetically closed in this experiment, and were placed inside a walk-in, temperature-regulated cold room maintained at 10 °C for 21 days. To establish the extent to which the VUV lamp heat penetrated the internal pulp temperature of the VUV exposed fruit were measured at sampling intervals, by inserting a thermosensor (TFX410 Ebro, Xylem Analytics, Germany) into the core of the fruit for about 60 s for a stable reading. Temperature deviation inside the fruit with VUV lamps (10 ± 1.85 °C) was comparable to the control setup. Generally, for every 10 °C rise in temperature, a 2 to threefold increase in biological reactions, such as respiration is expected (Caleb et al. 2012). Hence, heat due to VUV lamps inside chamber was considered negligible. On day 14, the VUV lights were switched off and the storage containers were opened, and the experiment was allowed to continue until day 21. Each set up was conducted in triplicate.

### Post-storage ethylene production and respiration rate

For samples exposed to VUV light and control, ethylene production rate (EPR) and respiration of apples was monitored on each sampling day. Apples were taken out of storage and allowed to acclimatize for an hour at ambient temperature. Samples were then placed in glass jars (3 L) and sealed hermetically. Concentration of ethylene was measured at regular intervals using an ICA 56-ethylene analyzer (Fricaval 89 S.L, Valencia, Spain). The ethylene production rate was calculated as the amount of ethylene produced per unit mass of the fruit per unit time (µL/kg h).

Respiration rate (RR) of apples was determined by placing a known mass from the treatment and control chamber into a closed system respirometer (developed in-house), which consisted of three glass jars fitted with tubes. Hermetic sealing was achieved with O-rings between the lid and the glass jar. Gas samples (CO_2_) were taken after 1 h using a gas analyzer (Oxycarb 6, Isolcell, Laives, Italy). The RR was calculated as the amount of CO_2_ produced per unit mass of the fruit per unit time (mL/kg h) using Eq. (3):

3 $$R_{{{\text{CO}}_{2} }} = \left( {Y_{{{\text{CO}}_{2} tf}} - Y_{{{\text{CO}}_{2} ti}} /\Delta t} \right)V_{f} /W$$

where $Y_{CO2 tf}$ and $Y_{CO2 ti}$ are CO2 concentration (%) at time $t_{f}$ (h) and time $t_{i}$ (h), respectively. $R_{{{\text{CO}}_{2} }}$ is RR due to CO2 production in mL/g h, Vf is the free volume of the containers (mL), and W is the total mass of the product (kg). All measurements were conducted in triplicate.

### Texture profile

Fruit tissue strength (hardness) of apples exposed to VUV and control was determined as the maximum force required to penetrate the tissue of peeled fruit using a texture analyzer (FTA 20, Güss, South Africa). Opposite sides (left, right) of the apple were gently peeled, placed on the platform and a 7.9 mm compression probe was used on each of the sides with a penetration distance of 8.9 mm and a speed of 10 mm/s. All measurements were conducted in triplicate per treatment and tissue strength was expressed in kg.

### Colour

Colour changes on each apple fruit exposed to VUV and control were measured based on Commission International del’ Eclairage (CIE) colour system using a digital Chroma-meter (CR 400/410 Konica Minolta Sensing Inc., Japan). Colour calibration of the chroma-meter was performed against a white and black tile background before each measurement. Colour measurements were taken using individual fruit $\left( {n = 6} \right)$ and data obtained were average of individual colour parameters. To describe the measured colour attributes hue angle $\left( {h^{0} } \right)$, which describes the qualitative attribute of colour shades (0° red–purple and 180° bluish-green), and Chroma (C*), which denotes the quantitative attribute of colour intensity were calculated using Eqs. (4) and (5):

4 $$h^{0} = tan^{ - 1} \left( {\frac{{b^{*} }}{{a^{*} }}} \right)$$

5 $$C^{*} = \sqrt {\left( {a^{*} } \right)^{2} + \left( {b^{*} } \right)^{2} }$$

where, L^*^ denotes the lightness, $a^{*}$ describes red ( +)/green (−) and $b^{*}$ describes yellow ( +)/blue (−).

### Total soluble solid (TSS) and titratable acidity (TA)

Fruit exposed to VUV and control were processed into juice using a juice extractor (4294 J700, Braun, China), and the juice obtained was used to measure total soluble solids (TSS) and titratable acidity (TA). Total soluble solid was measured using a calibrated pocket refractometer (PAL-1, ATAGO, Japan) and the results were expressed as °Brix. Titratable acidity of each fruit was obtained via titration of 53.7 mL of each fruit juice with 0.33 N of sodium hydroxide (NaOH) at a pH of 8.2, using Crison Titromatic 1S/2B (Crison Instruments, Barcelona, Spain) and the results were expressed as g/100 mL malic acid.

### Statistical analysis

Factorial analysis of variance (ANOVA) was used to elucidate the impacts of experimental factors/treatment and storage duration on measured quality parameters at 95% confident interval using Statistica Software vr. 13 (TIBCO, StatSoft Inc., Tulsa, OK, USA). Duncan multiple range test was used to determine the difference between mean values. All analyses were conducted in triplicate and results were presented as mean (*n* = 3) ± standard deviation.

## Results and discussion

### Ethylene degradation kinetics

**Fig. 2:**
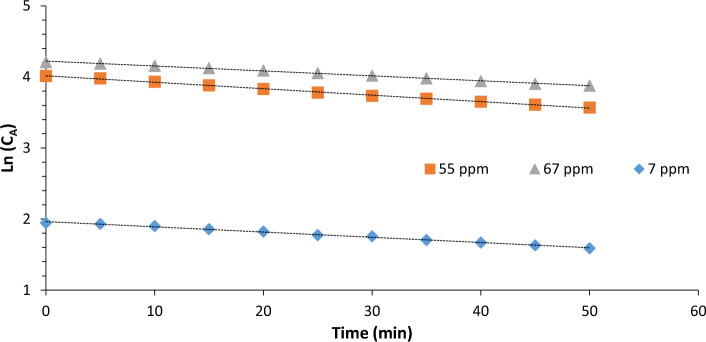
Degradation kinetics of ethylene fitted by the first-order kinetic model in the VUV reactor batch experiments. Experimental conditions: lamp power 3 W; initial concentrations of ethylene = 7, 55 and 67 ppm; duration 50 min

Degradation kinetics of ethylene by the VUV photolysis reactor at different initial concentrations were well fitted by the first-order kinetic model, and the observed rate constants are shown in Fig. 2. Removal of ethylene increased from 15.9% to 35.9% with an increase in initial concentration from 7 to 55 mg/kg but declined to 28.2% with a further increase to 67 mg/kg (Fig. 2). Similarly, the rate constant increased from 0.003/min to 0.0091/min when the initial ethylene concentration was increased from 7 to 55 mg/kg (Table 1). Increasing the concentration from 7 to 55 mg/kg resulted in more ethylene molecules interacting with the generated photons and hence resulted in higher conversion efficiency.

**Table 1:** Ethylene concentrations and estimated first order reaction rate constants obtained for VUV photolysis

Ethylene con. (ppm)	Ethylene removal (%)	Rate constant (min^−1^)	*R*^*2*^
7	15.9 ± 0.83^C^	0.0036	0.9944
55	35.9 ± 1.53^A^	0.0091	0.9979
67	28.2 ± 1.36^B^	0.0069	0.9972

On the other hand, a further increase of ethylene concentration from 55 to 67 mg/kg resulted in a decrease in the percentage removal and rate constant. This is attributed to the fact that since the number and energy of photons did not change, ethylene molecules obtained less energy as inlet concentration increased resulting in a low rate constant and percentage removal (Mabusela et al. 2021a). Additionally, high contents of ethylene might suppress the transmission of 185 nm UV light, thereby reducing the production of the HO^●^ radicals responsible for ethylene oxidation (Gómez Pacheco et al. 2012, Yao et al. 2016). These results are in accordance with the work of Chang et al. (2013) where the authors reported a decrease in ethylene percentage removal from 63 to 40% upon increasing initial concentration from 20 to 100 mg/kg. The removal of ethylene by VUV photolysis can be said to be a first-order reaction that is dependent on the initial ethylene concentration. Increasing the ethylene concentration beyond the threshold concentration resulted in decreased percentage removal.

### Ethylene degradation in mixed-fruit storage container

**Fig. 3:**
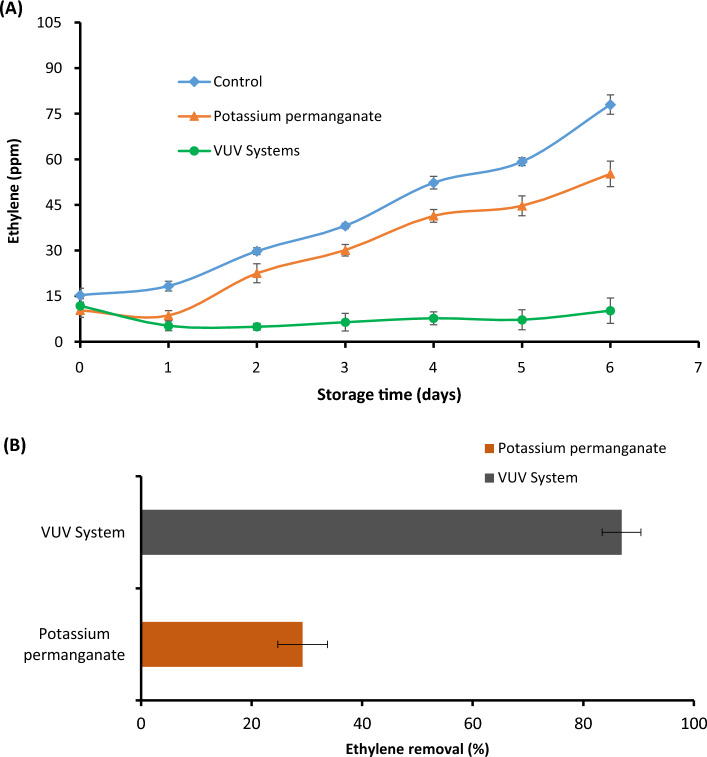
Changes in ethylene concentration in the different mixed-fruit storage chambers at ambient room temperature for 6 days **A**, and percentage ethylene removal in storage chambers connected with VUV system and potassium permanganate (industry practice) **B**

The change in ethylene concentration during the storage period under the different treatments containing mixed-fruit and the corresponding percentage of ethylene removal is shown in Fig. 3. The VUV system was able to significantly maintain the lowest ethylene concentration throughout the storage period. In contrast, the ethylene concentration continued to increase in the control and industry practice chambers to values of 78 and 58 mg/kg, respectively (Fig. 3A). The ethylene concentration accumulated in the control and industry practice chamber is sufficient to produce premature fruit ripening (Keller et al. 2013; Basso et al. 2018). Furthermore, the results indicated that ethylene percentage removal was higher in the storage container connected with the VUV photolysis reactor throughout the storage duration. By the end of the storage duration (day 6), the ethylene concentration reduced by 25% for fruits stored under KMnO_4_ and by 86.9% for fruits stored under the storage container with VUV photolysis reactor compared to the control fruit (Fig. 3B). The results obtained in this study are in agreement with the results reported by Pathak et al. (2019). In their study, a percentage removal of 96.28% from a storage chamber of apples connected to a VUV photolysis reactor was reported.

Potassium permanganate is a well-known ethylene scavenger and has been reported to show high ethylene removal. It is reported that KMnO_4_-based C_2_H_4_ absorbers have higher ethylene removal rates when supported onto nano-materials (Spricigo et al. 2017). However, no additional support material was used in this study with KMnO_4_, which could have resulted in lower ethylene removal rate compared to the VUV system. It is also suggested that the most suitable position to place the absorbent is the upper part of cold storage facility, since ethylene gas tends to rise to the top of the package due to it being less dense than air (Álvarez-Hernández et al. 2019), and in our study the absorbent tray was placed at the bottom of the storage container. However, it is noteworthy that ethylene was continuously removed by KMnO_4_ salt treatment as storage progressed (Fig. 3A), indicating that saturation level was not reached, and proportion used (10% kg/kg of fruit) was sufficient to bring down ethylene concentration. The VUV photolysis performed better than potassium permanganate because the reaction in KMnO_4_ salt occurs mainly on the surface, which became saturated over time, whereas photolysis occurs in the gas phase and therefore is faster resulting in higher percentage removal. The results from this study show that VUV photolysis could be a great alternative tool for ethylene removal in mixed-storage facilities or -loaded delivery truck.

## Changes associated with exposure of apples alone to direct VUV

### Post-storage respiration and ethylene production rate

**Fig. 4:**
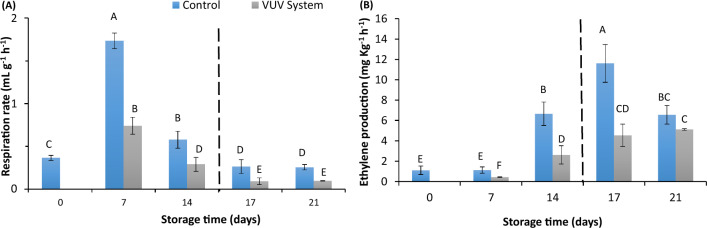
Post-storage respiration and ethylene production rate for ‘Fuji’ apples exposed to direct VUV and stored at 10 °C for 21 days. The VUV lamps were switched off on day 14 and the storage chambers were left opened until day 21

Respiration rate (RR) of apples in the control chamber was significantly higher compared to the RR of apples treated with direct VUV (Fig. 4A). This is response is consistent with the ethylene production rates reported in (Fig. 4B). Ethylene induces the respiratory burst of CO_2_ production in climacteric fruits and hence, the removal of ethylene by the VUV lamps resulted in low RR (Fagundes et al. 2015; Zagory 1995). On the contrary, the RR rate in both the control and treatment chamber decreased after day 14 when the storage containers were opened. The observed decrease in RRs in both storages is attributed to the fact that there was no longer accumulation effect of ethylene in both storage chambers as the storages were left open. The results from this study suggest that direct exposure of apples to VUV was able to reduce the RR in a closed storage. This demonstrates the potential of the system for maintaining fruit quality as low RR is associated with prolonged shelf-life.

Furthermore, the ethylene production rates of apples exposed to direct VUV is shown in Fig. 4B. The results show that the VUV lamp was able to suppress ethylene production during the 14 days of storage. The ethylene production rate of apples under VUV radiation was 2.62 µL/kg h on day 14, while a production rate of 6.66 µL/kg was achieved from apples in the control storage. When the VUV lamp was turned off, the production rate of ethylene increased by 42.4% after 3 days suggesting that the VUV lamp was responsible for retarding ethylene production. These results show that the direct exposure of apples to VUV light inhibited ethylene production during storage, which would subsequently delay fruit ripening. Similar results were achieved with UV-C light treatment for mangoes (Pristijono et al. 2018). During the direct exposure of apples, the hydroxyl radicals that are generated react quickly with the ethylene molecules and in such a setup, where the fruits are exposed to direct VUV light, the removal mechanism is by both direct and indirect photolysis (Huang et al. 2016b; Mabusela et al. 2021a). The results showed that apples exposed to direct VUV slowed endogenous ethylene production.

### Impact of direct VUV exposure on colour

Changes in colour parameters of ‘Fuji’ apples exposed to VUV and that of the control chamber is summarized in Table 2. Apples stored in the control chamber did not show a significant change in hue angle (*h°*). In contrast, the apples exposed to VUV radiation showed a significant (*p* < 0.05) decline in *h°* from 60.7 to 33.5. However, VUV radiation did not have a significant effect on the colour intensity (C^*^) and statistical difference were found between treatment and control. Furthermore, there was a significant decrease in the lightness (L^*^) parameter of ‘Fuji’ apples exposed to VUV radiation at the end of storage on day 21. Since a low value of L^*^ indicates a dark fruit skin, the results indicate that the apples exposed to VUV radiation were darker than the apples in the control.

**Table 2:** Changes in physical and biochemical quality attributes of ‘Fuji’ apples under direct exposure to VUV and control stored at 10 °C for 21 days

Treatment types	Storage durations	Quality parameter(s)					
		C*	*h°*	*L*	Firmness (kg)	Titratable acidity (g/100 mL)	Total soluble solids *(°Brix*)
Control	Day 0	35.5 ± 3.78^A^	60.7 ± 1.09^A^	58.1 ± 3.60^A^	6.8 ± 0.80^A^	0.58 ± 0.02^A^	14.2 ± 0.10^B^
	Day 7	33.7 ± 4.59^A^	64.3 ± 2.30^A^	54.5 ± 1.63^AB^	6.8 ± 0.80^A^	0.50 ± 0.01^B^	15.1 ± 0.18^A^
	Day 14	34.8 ± 2.16^A^	55.9 ± 7.92^A^	55.7 ± 2.20^AB^	6.7 ± 0.91^AB^	0.60 ± 0.02^A^	13.9 ± 0.66^B−D^
	Day 21	37.4 ± 2.76^A^	54.4 ± 10.50^A^	53.9 ± 0.6^B^	6.2 ± 0.62^AB^	0.49 ± 0.01^B^	14.4 ± 0.01^B^
VUV treatment	Day 0	35.5 ± 3.78^A^	60.7 ± 1.09^A^	58.1 ± 3.60^A^	6.8 ± 0.80^A^	0.58 ± 0.02^A^	14.2 ± 0.10^B^
	Day 7	31.3 ± 3.65^A^	58.8 ± 2.19^A^	50.3 ± 1.22^C^	6.2 ± 0.51^AB^	0.61 ± 0.04^A^	14.6 ± 0.30^AB^
	Day 14	32.3 ± 3.89^A^	66.46 ± 7.04^A^	49.8 ± 2.27^C^	6.4 ± 0.08^A^	0.47 ± 0.02^BC^	13.9 ± 0.06^C^
	Day 21	33.5 ± 1.07^A^	33.5 ± 9.51^B^	50.4 ± 1.13^C^	5.7 ± 0.24^B^	0.45 ± 0.01^C^	13.6 ± 0.11^D^

The value in the table are means and standard deviations. Different superscript letters in a column represent statistically significant differences (*p* ≤ 0.05) on the quality change at different storage time

Lourenço et al. (2016) investigated the effect of UV radiation treatment on the colour of papaya fruit at room temperature. The results showed that the fruit exposed to UV radiation had lower L^*^ value compared to untreated fruit. These observations agree with our results, meaning that the prolonged exposure of apples to VUV radiation accelerated chlorophyll degradation resulting in detrimental effects on the fruit appearance. Although it is reported that the degradation in fruit colour and ethylene production are correlated (Cheng et al. 2012), the loss in skin colour of apples in this study was attributed to ozone and the long-term exposure to VUV radiation since ethylene production was suppressed. These results suggest that ozone production in the storage chamber needs to be monitored and removed. This can be achieved by employing the use of ozone absorbers or catalysts.

### Impact of VUV exposure on fruit texture

The tissue strength of ‘Fuji’ apples in both treatments declined during storage (Table 2). Tissue strength of apples under VUV radiation was significantly (*p* ≤ 0.05) lower than those in the control. Apples under VUV radiation declined from the initial value of ≈ 6.8 kg on day 0 to 5.7 on day 21. Although texture profile of fruits can be correlated to the degraded ethylene during storage (Jia et al. 2020). In this study, VUV radiation was successful in removing ethylene, however, the loss in firmness of apples under VUV radiation reported could be attributed to the deterioration of cell wall by VUV radiation (Zhang and Jiang 2019).

On the other hand, the firmness of apples in the control did not change significantly although high ethylene accumulation was reported. This could be attributed to the effect of the cool temperature at which the study was conducted. The results from this study show that although direct VUV exposure was successful in removing the ethylene from storage, it had a negative impact on the firmness of apples. Therefore, the need for optimum system design is crucial for the application of VUV photolysis in storage facility.

### Total soluble solid (TSS) and titratable acidity (TA)

Total soluble solids (TSS) and titratable acidity (TA) of apples under VUV treatment and non-treated control is summarized in Table 2. Overall, TA declined significantly from 0.58 to 0.49 g/100 mL for apples in control storage chamber, and to 0.45 g/100 mL for apples under VUV radiation. Apples stored in the control chamber had relatively higher TA at the end of storage compared to the apples exposed to VUV light. This suggests that the apples stored under both control and the VUV light were physiologically stressed. The increase observed in CO_2_ and ethylene production rate observed in the control chamber may trigger the transformation of organic acids into sugars resulting in decreasing TA during apple ripening (Bruijn et al. 2019). The decrease in TA of apples exposed to VUV radiation could be attributed to the impact of direct VUV light around the fruit vicinity.

Furthermore, TSS content for apples in control fluctuated during the storage period with an initial increase from 14.2% to 15.1% at day 7 of storage and then decreased to 13.9% on day 14. The increase in TSS content during ripening of fruits and decrease after attaining peak levels is a result of natural fruit ripening and senescence processes that are typical of postharvest change (Siti Amirah et al. 2017). Similarly, the initial increase in TSS of apples in the control chamber is attributed to the presence of accumulated ethylene causing ripening, which increases sugar content. The increase in TSS of apples under VUV radiation was from 14.2% to 14.6% on day 7 and then declined to 13.6% on day 21. The results from this study show that VUV radiation in proximity had detrimental effect in maintaining TSS and TA of apples.

### Impact of direct VUV exposure on visual quality

**Fig. 5:**
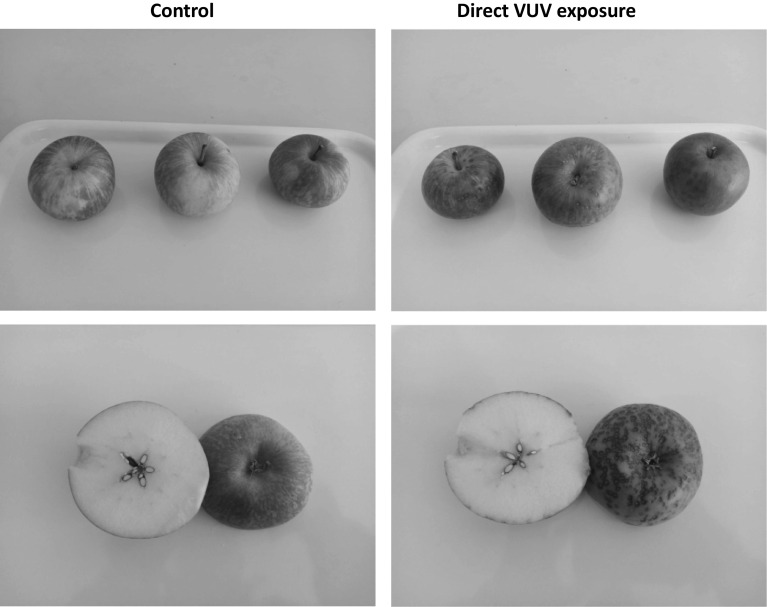
Effect of direct exposure of ‘Fuji’ apples to VUV radiation, and control treatments on the appearance and longitudinal section photos of apples after 21 days storage at 10 °C

Visual changes for ‘Fuji’ apples at the end of day 21 is presented in Fig. 5. Based on visual observation there was no damage on the skin surface of apples in the control storage chambers. On the contrary, the treatment with direct exposure to VUV radiation produced alterations in the skin colour of apples resulting in dark spots (Fig. 5). This was evident from the colour parameter data obtained and presented in Table 2. Similar observations were reported by Lourenço et al. (2016) where the authors found that the skin of papaya exposed to radiation was darker than the fruit in control fruit. The discoloration of fruit during exposure to UV treatments has been associated with the type of radiation, light induced anthocyanin biosynthesis (Zhao et al. 2017), and overdose of radiation (Ding et al. 2014), that could lead to breakage of cellular membrane, increase enzyme -substrate contact and led to tissue color change (Sethi et al. 2018). Direct exposure of ‘Fuji’ apples to VUV/UV during storage or retail display as a measure of controlling ethylene biosynthesis is not a good practice and not encouraged. Thus, it is important to design an integrated ethylene scavenging-reactor system with storage facility to prevent/minimize direct fruit contact with UV radiation and other reactive species.

Furthermore, vacuum ultraviolet photolysis results in the generation of many different reactive oxygen species, such as hydroxyl radicals and ozone (Mabusela et al. 2021a). It can be inferred that ozone could be responsible for damaging the skin colour of the apples. Jia et al. (2020) also noticed symptoms of injury and pitted structures on the skin of peaches caused by ozone. Although the application of direct VUV radiation was successful in suppressing the production of ethylene, an undesirable effect of the VUV radiation on the appearance of apples was observed. This could be avoided by reducing the exposure time and by coupling the technique with ozone absorbers to eliminate the residual ozone. Furthermore, when employing VUV photolysis technique for postharvest management, the produce should not be directly exposed to radiation to prevent loss in fruit quality.

## Conclusion

This study investigated the potential of a VUV photolysis reactor for the removal of ethylene during the storage of mixed-fruit. Also, the effect of direct VUV exposure on the physicochemical properties of apples was investigated. According to the results, the kinetics of ethylene degradation by VUV photolysis was found to follow a first-order kinetic model. It was also shown that the VUV reactor was able to reduce ethylene concentration in the mixed-fruit storage container by 86.9% compared to 47% achieved by KMnO_4_. Therefore, a closed air circulation VUV system connected to the storage chamber is a promising technique for ethylene removal in the storage environment of mixed-fruit and could offer a better solution in maintaining the postharvest quality of fruit. The response of the ‘Fuji’ apples to direct exposure of VUV radiation could be a function of cultivar sensitivity and/or selectivity, thus other apple cultivars should be investigated for comparison.

Furthermore, the findings showed that for the application of VUV photolysis in postharvest management of ethylene biosynthesis, direct exposure to horticultural commodity is not recommended to avoid deteriorative impact of VUV radiation on fruit quality. The impact of heat generated by the VUV lamp on storage temperature around the fruit was not monitored in this study. Hence, future studies should incorporate light and temperature sensors inside the storage chamber, and focus on the optimization of VUV photo intensity, free-volume, and distance between the lamp and fruit during storage. Lastly, the VUV modular reactor should be integrated with temperature sensor and an ozone scrubber to ensure removal of residual ozone and that the direct exposure of apples to VUV radiation is prevented by using an air circulation system.

## Data Availability

The data that support the findings of this study is available from the corresponding author on request.

## References

[CR1] Álvarez-Hernández MH, Martínez-Hernández GB, Avalos-Belmontes F, Castillo-Campohermoso MA, Contreras-Esquivel JC, Artés-Hernández F (2019). Potassium permanganate-based ethylene scavengers for fresh horticultural produce as an active packaging. Food Eng Rev.

[CR2] Aprilliani F, Warsiki E, Iskandar A (2018) Kinetic studies of potassium permanganate adsorption by activated carbon and its ability as ethylene oxidation material. In: IOP conference series: earth environmental science, p 012003. 10.1088/1755-1315/141/1/012003

[CR3] Basso A, Moreira RDFPM, José HJ (2018). Effect of operational conditions on photocatalytic ethylene degradation applied to control tomato ripening. J Photochem Photobiol A Chem.

[CR4] Bruij JD, Gomez A, Melin P, Loyola C, Solar VA, Valdés H (2019). Effect of doping natural zeolite with copper and zinc cations on ethylene removal and postharvest tomato fruit quality. Chem Eng Trans.

[CR5] Caleb OJ, Mahajan PV, Opara UL, Witthuhn CR (2012). Modelling the effect of time and temperature on respiration rate of pomegranate arils (cv. ‘Acco’ and ‘Herskawitz’). J Food Sci.

[CR6] Chang KL, Sekiguchi K, Wang Q, Zhao F (2013). Removal of ethylene and secondary organic aerosols using UV-C254 + 185 nm with TiO_2_ catalyst. Aerosol Air Qual Res.

[CR7] Cheng Y, Dong Y, Yan H, Ge W, Shen C, Guan J, Liu L, Zhang Y (2012). Effects of 1-MCP on chlorophyll degradation pathway-associated genes expression and chloroplast ultrastructure during the peel yellowing of Chinese pear fruits in storage. Food Chem.

[CR8] Ding P, Ling YS (2014). Browning assessment methods and polyphenol oxidase in UV-C irradiated Berangan banana fruit. Int Food Res J.

[CR9] Duque LF, Amador MV, Guzmán M, Asensio C, Valenzuela JL (2021). Development of a new essential oil-based technology to maintain fruit quality in tomato. Hortic.

[CR10] Fagundes C, Moraes K, Pérez-Gago MB, Palou L, Maraschin M, Monteiro AR (2015). Effect of active modified atmosphere and cold storage on the postharvest quality of cherry tomatoes. Postharvest Biol Technol.

[CR11] Gómez Pacheco C, Sánchez-Polo M, Rivera-Utrilla J, López-Peñalver J (2012). Tetracycline degradation in aqueous phase by ultraviolet radiation. Chem Eng J.

[CR12] Huang H, Huang H, Zhan Y, Liu G, Wang X, Lu XL, Feng Q, Leung DYC (2016). Efficient degradation of gaseous benzene by VUV photolysis combined with ozone-assisted catalytic oxidation: performance and mechanism. Appl Catal B Environ.

[CR13] Huang H, Lu H, Huang H, Wang L, Jieni Z, Leung DYC (2016). Recent development of VUV-based processes for air pollutant degradation. Front Environ Sci.

[CR14] Jia X, Li J, Du M, Zhao Z, Song J, Yang W, Zheng Y, Chen L, Li X (2020). Combination of low fluctuation of temperature with TiO_2_ photocatalytic/ozone for the quality maintenance of postharvest peach. Foods.

[CR15] Kang IS, Xi J, Hu HY (2018). Photolysis and photooxidation of typical gaseous VOCs by UV Irradiation: Removal performance and mechanisms. Front Environ Sci Eng.

[CR16] Keller N, Ducamp MN, Robert D, Keller V (2013). Ethylene removal and fresh product storage: a challenge at the frontiers of chemistry. toward an approach by photocatalytic oxidation. Chem Rev.

[CR17] Lourenço RERS, Linhares AAN, Oliveira AVD, Silva MGD, Oliveira JGD, Canela MC (2016). Photodegradation of ethylene by use of TiO_2_ sol-gel on polypropylene and on glass for application in the postharvest of papaya fruit. Environ Sci Pollut Res Int.

[CR18] Mabusela B, Belay ZA, Godongwana B, Pathak N, Mahajan PV, Caleb OJ (2021). Advances in vacuum ultraviolet photolysis in the postharvest management of fruit and vegetables along the value chains: a review. Food Bioprocess Technol.

[CR19] Mabusela B, Belay ZA, Godongwana B, Pathak N, Mahajan PV, Mathabe PMK, Caleb OJ (2021). Trends in ethylene management strategies: towards mitigating postharvest losses along the South African value chain of fresh produce–a review. S Afr J Plant Soil.

[CR20] Mahmoudkhani F, Rezaei M, Asili V, Atyabi M, Vaisman E, Langford CH, Visscher AD (2016). Benzene degradation in waste gas by photolysis and photolysis-ozonation: experiments and modeling. Front Environ Sci Eng.

[CR21] Mortazavian S, Saber A, James DE (2019). Optimization of photocatalytic degradation of acid blue 113 and acid red 88 textile dyes in a UV-C/TiO_2_ suspension system: application of response surface methodology (RSM). Catalysts.

[CR22] Palou LS, Crisosto CH, Garner D, Basinal LM (2003). Effect of continuous exposure to exogenous ethylene during cold storage on postharvest decay development and quality attributes of stone fruits and table grapes. Postharvest Biol Technol.

[CR23] Pathak N, Caleb OJ, Geyer M, Herppich WB, Rauh C, Mahajan PV (2017). Photocatalytic and photochemical oxidation of ethylene: potential for storage of fresh produce—a review. Food Bioprocess Technol.

[CR24] Pathak N, Caleb OJ, Rauh C, Mahajan PV (2017). Effect of process variables on ethylene removal by vacuum ultraviolet radiation: application in fresh produce storage. Biosyst Eng.

[CR25] Pathak N, Caleb OJ, Rauhc C, Mahajan PV (2019). Efficacy of photocatalysis and photolysis systems for the removal of ethylene under different storage conditions. Posthavest Biol Technol.

[CR26] Pristijono P, Golding JB, Bowyer MC (2018). Postharvest UV-C treatment, followed by storage in a continuous low-level ethylene atmosphere, maintains the quality of ‘Kensington Pride’ mango fruit stored at 20 °C. Hortic.

[CR27] Sethi S, Joshi A, Arora B (2018). UV treatment of fresh fruits and vegetables. Postharvest disinfection of fruits and vegetables.

[CR28] Siti Amirah MZ, Nor Afifah AR, Husni Hayati MR, Wan Zaliha WS (2017) The effects of charcoal from different agricultural wastes in reducing ethylene production of Berangan Banana (*Musa* sp. AAA Berangan). In: Proceedings of the international conference of FoSSA Jember, p 201–210

[CR29] Smilanick JL (2003) Use of ozone in storage and packing facilities. In: Paper presented at the Washington tree fruit postharvest conference

[CR30] Spricigo PC, Foschini MM, Ribeiro C, Corrêa DS, Ferreira MD (2017). Nanoscaled platforms based on SiO_2_ and Al_2_O_3_ impregnated with potassium permanganate use color changes to indicate ethylene removal. Food Bioprocess Technol.

[CR31] Yao H, Pei J, Wang H, Fu J (2016). Effect of Fe(II/III) on tetracycline degradation under UV/VUV irradiation. Chem Eng J.

[CR32] Zagory D, Rooney ML (1995). Ethylene-removing packaging. Active food packaging.

[CR33] Zhang W, Jiang W (2019). UV treatment improved the quality of postharvest fruits and vegetables by inducing resistance. Trends Food Sci Technol.

[CR34] Zhao Y, Dong W, Wang K, Zhang B, Allan AC, Lin-Wang K, Xu C (2017). Differential sensitivity of fruit pigmentation to ultraviolet light between two peach cultivars. Front Plant Sci.

[CR35] Zhu Z, Zhang Y, Shang Y, Wen Y (2019). Electrospun nanofibers containing TiO_2_ for the photocatalytic degradation of ethylene and delaying postharvest ripening of bananas. Food Bioprocess Technol.

